# Curving the space by non-Hermiticity

**DOI:** 10.1038/s41467-022-29774-8

**Published:** 2022-04-21

**Authors:** Chenwei Lv, Ren Zhang, Zhengzheng Zhai, Qi Zhou

**Affiliations:** 1 Department of Physics and Astronomy, Purdue University, West Lafayette, IN 47907 USA; 2 School of Physics, Xi’an Jiaotong University, Xi’an, Shaanxi 710049 China; 3 Purdue Quantum Science and Engineering Institute, Purdue University, West Lafayette, IN 47907 USA

**Keywords:** Ultracold gases, Topological matter, Quantum optics

## Abstract

Quantum systems are often classified into Hermitian and non-Hermitian ones. Extraordinary non-Hermitian phenomena, ranging from the non-Hermitian skin effect to the supersensitivity to boundary conditions, have been widely explored. Whereas these intriguing phenomena have been considered peculiar to non-Hermitian systems, we show that they can be naturally explained by a duality between non-Hermitian models in flat spaces and their counterparts, which could be Hermitian, in curved spaces. For instance, prototypical one-dimensional (1D) chains with uniform chiral tunnelings are equivalent to their duals in two-dimensional (2D) hyperbolic spaces with or without magnetic fields, and non-uniform tunnelings could further tailor local curvatures. Such a duality unfolds deep geometric roots of non-Hermitian phenomena, delivers an unprecedented routine connecting Hermitian and non-Hermitian physics, and gives rise to a theoretical perspective reformulating our understandings of curvatures and distance. In practice, it provides experimentalists with a powerful two-fold application, using non-Hermiticity to engineer curvatures or implementing synthetic curved spaces to explore non-Hermitian quantum physics.

## Abstract (web summary)

The understanding and control of non-Hermitian phenomena is becoming every day more important. Here the authors establish the duality between non-Hermiticity and curved spaces. It unfolds a geometric root of non-Hermitian phenomena and provides a study and tailor non-Hermiticity using curved spaces.

## Introduction

System-environment couplings lead to a plethora of intriguing non-Hermitian phenomena^1–9^, such as non-orthogonal eigenstates, the non-Hermitian skin effect^10–16^, real energy spectra in certain parameter regimes^17,18^, collapsed energy spectra, and coalesced eigenstates at an exceptional point (EP)^12,19,20^, and drastic responses to boundary conditions^21,22^. While these phenomena have been extensively explored in quantum sciences and technologies^2–5,8,23–27^, peculiar theoretical tools are often required to study non-Hermitian physics. Though bi-orthogonal vectors and metric operators are introduced to restore orthogonality^11,19,28–30^, the underlying physics of these mathematical tools is not clear yet. Moreover, it remains challenging to prove the real energy spectra of certain non-Hermitian systems, as the existence of the ${{{{{{{\mathcal{PT}}}}}}}}$ symmetry does not guarantee a real energy spectrum and sophisticated mathematical techniques are required^17,18,31^.

In this work, we show a duality between non-Hermitian Hamiltonians in flat spaces and their counterparts in curved spaces. On the theoretical side, this duality leads to a geometric framework providing a unified explanation of several non-Hermitian phenomena. For instance, it is the finite curvature that requires an orthonormal condition distinct from that in flat spaces, enforces eigenstates to localize at edges, and gives rise to the supersensitivity to boundary conditions. Dual models in curved spaces could be Hermitian, providing a simple proof of the existence of real energy spectra in certain non-Hermitian systems. Moreover, in sharp contrast to existing schemes of studying curved spaces^32–35^, which were built on the conventional wisdom that a flat space needs to be physically distorted to become curved, our results show that non-Hermiticity is a controllable knob for tuning curvatures even when the space appears to be flat, for instance, in lattices with fixed lattice spacing. This duality therefore may reform our understanding of distance and curvatures.

In practice, our duality has a two-fold implication. On the one hand, it establishes non-Hermiticity as a unique tool to simulate intriguing quantum systems in curved spaces. For instance, it offers an approach of using non-Hermitian systems in flat spaces to solve the grand challenge of accessing gravitational responses of quantum Hall states (QHS) in curved spaces^34,36–38^. On the other hand, the duality allows experimentalists to use curved spaces to explore non-Hermitian physics. Whereas a variety of non-Hermitian phenomena have been addressed in experiments, delicate designs of dissipations are often required^2,4,7–9,39–42^. Our results show that curved spaces can serve as an unprecedented means to explore non-Hermitian Hamiltonians without resorting to dissipations.

## Results

### Hatano-Nelson model and hyperbolic surfaces

Our duality can be demonstrated using the celebrated Hatano-Nelson (HN) model^43^, which reads,

1 $$-{t}_{R}{\psi }_{n-1}-{t}_{L}{\psi }_{n+1}=E{\psi }_{n},$$

where *n* = 0, 1, . . . *N* − 1 is the lattice index of a one-dimensional (1D) chain, *ψ*_*n*_ is the eigenstate, *E* is the corresponding eigenenergy, and *t*_*L*_ and *t*_*R*_ are the nearest-neighbor tunneling amplitudes towards the left and the right, respectively. Under the open boundary condition (OBC), *ψ*_0_ = *ψ*_*N*−1_ = 0, ${\psi }_{n}={e}^{n\ln (\gamma )}\sin ({k}_{m}nd)/\sqrt{(N-1)/2}$, where $\gamma =\sqrt{{t}_{R}/{t}_{L}}$ characterizes the strength of non-Hermiticity, *k*_*m*_ = *mπ*/((*N* − 1)*d*), *m* = 1, 2, . . . *N* − 2, and *d* is the lattice constant. The eigenenergy reads ${E}_{m}=-2\sqrt{{t}_{L}{t}_{R}}\cos ({k}_{m}d)$.

**Fig. 1:**
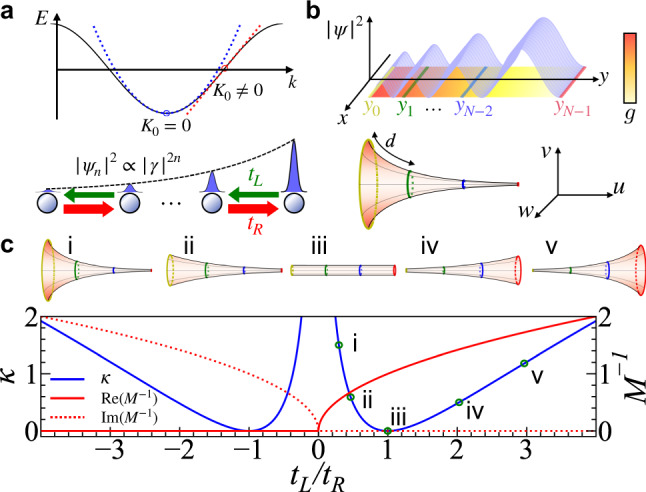
The duality between the Hatano-Nelson (HN) model and a hyperbolic surface. **a** A HN chain and its energy spectrum as a function of *k*. Near a vanishing (finite) *K*_0_, the effective theory in curved space is non-relativistic (relativistic). Eigenstates on the HN chain are localized at the edge, ∣*ψ*_*n*_∣^2^ ∝ ∣*γ*∣^2*n*^. **b** A HN chain is mapped to the shaded strip on the Poincaré half-plane, in which an eigenstate with *k*_*x*_ = 0 satisfies ∣*ψ*∣^2^ ∝ *y*. This shaded strip on the Poincaré half-plane with PBC in the *x*-direction is equivalent to a pseudosphere embedded in 3D Euclidean space. **c** The curvature and the inverse of the effective mass, as functions of *t*_*L*_ for a fixed *t*_*R*_. The unites of *κ* and *M*^−1^ are 1/*d*^2^ and 2*t*_*R*_*d*^2^/(ℏ^2^), respectively. (i–v) show the dual pseudospheres of the HN model at various *t*_*L*_ > 0. A pseudosphere for *t*_*L*_ < 0 is the same as that for −*t*_*L*_.

Similar to Hermitian lattice models, the effective theory of Eq. (1) in the continuum limit describes the motion of a non-relativistic (relativistic) particle at (away from) the band bottom and top, with a quadratic (linear) dispersion relation, as shown in Fig. 1a. At the band bottom, the effective theory is written as

2 $$-\frac{{\hslash }^{2}}{2M}\kappa \left({y}^{2}{\partial }_{y}^{2}+\frac{1}{4}\right)\psi (y)=E\psi (y),$$

where $M={\hslash }^{2}/(2\sqrt{{t}_{L}{t}_{R}}{d}^{2})$ is the effective mass, and $\kappa =4{\ln }^{2}(| \gamma | )/{d}^{2}$. Solutions to Eq. (2), ${y}^{\frac{1}{2}}{y}^{\pm i{k}_{y}/\sqrt{\kappa }}$, have the same energy, ${\hslash }^{2}{k}_{y}^{2}/(2M)$. An eigenstate under OBC is their superposition, $\psi (y)=\sqrt{2/\ln ({y}_{N-1}/{y}_{0})}{(y/{y}_{0})}^{\frac{1}{2}}\sin \left[{k}_{y}\ln (y/{y}_{0})/\sqrt{\kappa }\right]$ with ${k}_{y}=m\pi \sqrt{\kappa }/\ln ({y}_{N-1}/{y}_{0})$ and *ψ*(*y*_0_) = *ψ*(*y*_*N*−1_) = 0. *y*_0_ and *y*_*N*−1_ specify the positions of the two edges. At the band top, we have *M* → − *M*. Eq. (2) is a dimension reduction of the Schrödinger equation on a Poincaré half-plane,

3 $$-\frac{{\hslash }^{2}}{2M}\kappa \left({y}^{2}{\nabla }^{2}+\frac{1}{4}\right){{\Psi }}(x,y)=E{{\Psi }}(x,y),$$

where ${\nabla }^{2}\equiv \left({\partial }_{x}^{2}+{\partial }_{y}^{2}\right)$, ${{\Psi }}(x,y)={e}^{i{k}_{x}x}\psi (y)$, and − *κ* is the curvature (Supplementary Material). The metric tensor is ${{{{{{{\bf{g}}}}}}}}=\frac{1}{\kappa {y}^{2}}({{{{{{{\rm{d}}}}}}}}{x}^{2}+{{{{{{{\rm{d}}}}}}}}{y}^{2})$, $g=\det ({{{{{{{\bf{g}}}}}}}})=1/({\kappa }^{2}{y}^{4})$. Since *k*_*x*_ is a good quantum number, Eq. (3) reduces to Eq. (2) when *k*_*x*_ = 0. A finite *k*_*x*_ adds an onsite potential to the HN model,

4 $$V_{n}{\psi }_{n}-{t}_{R}{\psi }_{n-1}-{t}_{L}{\psi }_{n+1}=E{\psi }_{n},$$

where $V_{n}={a}^{2}\sqrt{{t}_{R}{t}_{L}}{\gamma }^{4n}$. The dimensionless quantity ${a}^{2}=4({\ln }^{2}| \gamma | ){y}_{0}^{2}{k}_{x}^{2}$ characterizes the strength of *V*_*n*_.

To derive the duality between the continuum limit of Eq. (1) at the band bottom and Eq. (2), we define ${\psi }_{n}\equiv \sqrt{d}\psi ({s}_{n})$ with *s*_*n*_ = *nd*, such that the eigenstate of the HN model, *ψ*_*n*_, defined on discrete lattice sites is extended to *ψ*(*s*) as a function of a continuous variable *s*. Since *ψ*_*n*_, under OBC, includes a part that changes exponentially, i.e., ${e}^{n\ln (\gamma )}$, so does *ψ*. We thus define *ϕ*(*s*) ≡ *ψ*(*s*)*e*^−*qs*^ with $q=\ln (\gamma )/d=\frac{1}{2d}\ln ({t}_{R}/{t}_{L})$ determining the inverse of the localization length, and *ϕ*(*s*) varies slowly with changing *s*. Then we have ${\psi }_{n}=\sqrt{d}\phi ({s}_{n}){e}^{q{s}_{n}}$. Substituting *ψ*_*n*_ into Eq. (1) and using the Taylor expansion for *ϕ*(*s*), $\phi ({s}_{n\pm 1})=\phi ({s}_{n})\pm d{\partial }_{s}\phi +\frac{1}{2}{d}^{2}{\partial }_{s}^{2}\phi$, we obtain $-\sqrt{{t}_{L}{t}_{R}}(2+{\partial }_{s}^{2})\phi =E\phi$. Consequently, *ψ*(*s*) satisfies

5 $$-\sqrt{{t}_{L}{t}_{R}}{d}^{2}\left({\partial }_{s}^{2}-2q{\partial }_{s}+{q}^{2}+2/{d}^{2}\right)\psi (s)=E\psi (s).$$

It describes a nonrelativistic particle subject to an imaginary vector potential, *A* ~ *iq*^39,43^. Unlike a real vector potential that amounts to a *U*(1) gauge field, here, an imaginary vector potential curves the space. Performing a coordinate transformation *y*/*y*_0_ = *e*^2*qs*^ and applying $M={\hslash }^{2}/(2\sqrt{{t}_{L}{t}_{R}}{d}^{2})$, $\kappa =4{\ln }^{2}(| \gamma | )/{d}^{2}$, we obtain Eq. (2) up to a constant energy shift $-2\sqrt{{t}_{L}{t}_{R}}$. The mapping between these two models is summarized in Table 1, which provides a dictionary translating microscopic parameters between them. For instance, *ψ*_*n*_, the wavefunction at the *n*-th lattice site of the NH model is identical to *ψ*(*y*_*n*_), the wavefunction on the Poincaré half-plane evaluated at ${y}_{n}={y}_{0}{e}^{n\sqrt{\kappa }d}$. The low-energy limit of the eigenenergy of Eq. (1) is also identical to the eigenenergy of Eq. (2) as shown by Table 1.

**Table 1:** The mapping between the continuum limit of the HN model near the band bottom under OBC and the Poincaré half-plane.

2D Hyperbolic surface	Curvature − *κ*	Energy scales ℏ^2^/(2*Md*^2^)	Coordinate ${y}_{n}={y}_{0}{e}^{n\sqrt{\kappa }d}$	Eigenfunctions $\psi (y)\propto {(y/{y}_{0})}^{\frac{1}{2}}\sin \left(\frac{m\pi \ln (y/{y}_{0})}{\ln ({y}_{N-1}/{y}_{0})}\right)$	Eigenenergies ${E}_{m}=\frac{\kappa {\hslash }^{2}{m}^{2}{\pi }^{2}}{2M\ln {({y}_{N-1}/{y}_{0})}^{2}}$
1D Non-Hermitian chain	Non-Hermiticity $-4{\ln }^{2}(| \gamma | )/{d}^{2}$	Tunneling strength $\sqrt{{t}_{R}{t}_{L}}$	Lattice site *n*	${\psi }_{n}\propto {\gamma }^{n}\sin \left(\frac{m\pi }{N-1}n\right)$	$\frac{{E}_{m}}{2\sqrt{{t}_{R}{t}_{L}}}=\left(-1+\frac{{m}^{2}{\pi }^{2}}{2{(N-1)}^{2}}+O\left(\frac{{m}^{4}}{{N}^{4}}\right)\right)$

Away from the band bottom(top), similar calculations can be performed by defining $\psi (s)={e}^{\pm i{K}_{0}s}{e}^{qs}\phi (s)$ using Taylor expansions of the slowly varying *ϕ*(*s*) and the same coordinate transformation *y* = *y*_0_*e*^2*qs*^. We obtain the effective theory near *K*_0_*d* ≠ 0, ±*π*,

6 $$\big[E({K}_{0})\pm i\sqrt{\kappa }\hslash {v}_{F}y({\partial }_{y}-1/(2y))\big]\psi (y)=E\psi (y),$$

where $E({K}_{0})=-2\sqrt{{t}_{L}{t}_{R}}(\cos ({K}_{0}d)+{K}_{0}d\sin ({K}_{0}d))$, ${v}_{F}=-2\sqrt{{t}_{L}{t}_{R}}d\sin ({K}_{0}d)/\hslash$, and ± corresponds to the left and right moving waves centered near ± *K*_0_, respectively, The previously defined $\kappa =4{\ln }^{2}(| \gamma | )/{d}^{2}$ has been used. The eigenstate under OBC includes both the left and right moving waves and is written as $\sqrt{2/\ln ({y}_{N-1}/{y}_{0})}{(y/{y}_{0})}^{\frac{1}{2}}\sin \big[{k}_{y}\ln (y/{y}_{0})/\sqrt{\kappa }\big]$ with eigenenergy of $-2\sqrt{{t}_{L}{t}_{R}}[\cos ({K}_{0}d)+({K}_{0}-{k}_{y})d\sin ({K}_{0}d)]$, which recovers the results of the HN model near a finite *K*_0_.

### Geometric interpretations of non-Hermitian phenomena

Our duality provides a natural explanation of several peculiar non-Hermitian phenomena. Firstly, the orthonormal condition of effective theories in Eq. (2) and Eq. (6) reads

7 $$\int \frac{{{{{{{{\rm{d}}}}}}}}y}{\kappa {y}^{2}}{\psi }^{* }(y;{k}_{y})\psi (y;{k}_{y}^{\prime})={{{{{{{\mathcal{N}}}}}}}}{\delta }_{{k}_{y},{k}_{y}^{\prime}},$$

where the normalization constant ${{{{{{{\mathcal{N}}}}}}}}$ can be chosen freely. As a common feature of curved spaces, a finite curvature appears in the above equation. Considering a strip in the domain *x*_0 _≤ *x* ≤ *x*_0_ + *L*, its width in the *x*-direction depends on *y*, ${L}_{x}(y)=\int\nolimits_{x = {x}_{0}}^{{x}_{0}+L}{{{{{{{\rm{d}}}}}}}}x/(\sqrt{\kappa }y)=L/(\sqrt{\kappa }y)$. Thus, a wave packet traveling in the *y*-direction must include an extra factor ${y}^{\frac{1}{2}}$ to guarantee the conservation of particle numbers. In the *s*-coordinate, Eq. (7) is written as $\int \frac{{{{{{{{\rm{d}}}}}}}}s}{\sqrt{\kappa }{y}_{0}}{e}^{-2qs}{\psi }_{{k}_{y}}^{* }(s){\psi }_{{k}_{y}^{\prime}}(s)={{{{{{{\mathcal{N}}}}}}}}{\delta }_{{k}_{y},{k}_{y}^{\prime}}$. Discretizing this equation with ${{{{{{{\mathcal{N}}}}}}}}={(\sqrt{\kappa }{y}_{0})}^{-1}$, and transforming it to the HN model, we obtain,

8 $${\sum }_{n}| \gamma {| }^{-2n}{\psi }_{n}^{* }({k}_{m}){\psi }_{n}({k}_{{m}^{\prime}})={\delta }_{{k}_{m},{k}_{{m}^{\prime}}},$$

where ∣*γ*∣^−2*n*^ is precisely the difference between the left and right eigenvectors, or the metric operator^30^. The mapping to a curved space thus establishes an explicit physical interpretation of orthonormal conditions in non-Hermitian systems.

Secondly, the duality allows us to equate the non-Hermitian skin effect to its counterpart on the Poincaré half-plane we found recently^44^. This can be best visualized using the embedding of a hyperbolic surface in three-dimensional (3D) Euclidean space. We define $y={r}_{0}\cosh (\eta )$, *x* = *r*_0_*φ*, where *r*_0_ is an arbitrary constant and *η* > 0, *φ* ∈ (−*π*, *π*). The embedding can then be written as

9 $$(u,v,w)=\frac{1}{\sqrt{\kappa }}\left((\eta -\tanh (\eta )),\frac{\cos (\varphi )}{\cosh (\eta )},\frac{\sin (\varphi )}{\cosh (\eta )}\right).$$

This is a parameterization of a pseudosphere with a constant negative curvature and a radius of $1/\sqrt{\kappa }$, which satisfies ${(u-{{{{{{{\rm{arcsech}}}}}}}}(\sqrt{({v}^{2}+{w}^{2})\kappa })/\sqrt{\kappa })}^{2}+{v}^{2}+{w}^{2}={\kappa }^{-1}$. As shown in Fig. 1b, a pseudosphere features a funnel shape, since the circumference of the circle with a fixed *y*(*η*) changes with changing *y*(*η*). As previously explained, a coordinate transformation *y* = *y*_0_*e*^2*qs*^ maps eigenstates on the hyperbolic surface, ${y}^{\frac{1}{2}}{y}^{i{k}_{y}/\sqrt{\kappa }}$, to ${e}^{qs}{e}^{i(2q/\sqrt{\kappa }){k}_{y}s}$, which exponentially localizes near the funneling mouth, the smaller end.

Thirdly, the collapsed energy spectrum at EP of the HN models has a natural geometric interpretation. When *t*_*L*_ = *t*_*R*_, the pseudosphere reduces to a cylinder with a vanishing *κ*. For a given *t*_*R*_( > *t*_*L*_), *κ* increases with decreasing *t*_*L*_. Increasing the non-Hermiticity thus makes the space more curved, as shown by Fig. 1 redc. Approaching EP, *t*_*L*_ → 0, *κ* diverges, and the localization length, $1/\ln (| \gamma | )$, vanishes, forcing all eigenstates to coalesce. As eigenenergies read $E={\hslash }^{2}{k}_{y}^{2}/(2M)$ with divergent *M*, eigenenergies collapse to zero with a massive degeneracy. Across EP, *t*_*L*_*t*_*R*_ < 0, and the effective mass becomes imaginary, all previous results of positive *t*_*L*_*t*_*R*_ still apply provided that *M* → ±*iM*. Particles moving in hyperbolic spaces are thus dissipative, and stationary states no longer exist.

Lastly, similar to the HN model, changing OBC to PBC leads to drastic changes in the curved space. Eigenstates of Eq. (2) and Eq. (6) normalized to ${{{{{{{\mathcal{N}}}}}}}}$ become $\sqrt{\kappa {{{{{{{\mathcal{N}}}}}}}}}{({y}_{0}^{-1}-{y}_{N-1}^{-1})}^{-\frac{1}{2}}{(y/{y}_{0})}^{i{k}_{y}/\sqrt{\kappa }}$, where ${k}_{y}=2m\pi \sqrt{\kappa }/\ln ({y}_{N-1}/{y}_{0})$) so that *ψ*(*y*_0_) = *ψ*(*y*_*N*−1_). Correspondingly, eigenenergies become complex. This can be explicitly shown from the time-dependent Schrödinger equations. For instance, at the band bottom(top), we multiply ψ^*^(*y*) to both sides of $i\hslash {\partial }_{t}\psi =-\frac{{\hslash }^{2}\kappa }{2M}\left({y}^{2}{\partial }_{y}^{2}+1/4\right)\psi$, subtract from the resultant expression its complex conjugate, and integrate over *y* from *y*_0_ to *y*_*N*−1_. We find that the total particle number ${{{{{{{{\mathcal{N}}}}}}}}}_{p}=\int\nolimits_{{y}_{0}}^{{y}_{N-1}}{{{{{{{\rm{d}}}}}}}}y| \psi (y){| }^{2}/(\kappa {y}^{2})$ satisfies,

10 $${\partial }_{t}{{{{{{{{\mathcal{N}}}}}}}}}_{p}=\hslash \sqrt{\kappa }{{{{{{{\mathcal{N}}}}}}}}{k}_{y}/M,$$

which signifies the absence of a stationary state and explains complex eigenenergies under PBC. Using ${\partial }_{t}{{{{{{{{\mathcal{N}}}}}}}}}_{p}=\frac{2}{\hslash }{{{{{{{\rm{Im}}}}}}}}(E){{{{{{{{\mathcal{N}}}}}}}}}_{p}$, we find ${{{{{{{\rm{Im}}}}}}}}(E)={\hslash }^{2}\sqrt{\kappa }{k}_{y}/(2M)$. This is distinct from the result for OBC, where $\psi (y) \sim {y}^{1/2}{y}^{\pm i{k}_{y}/\sqrt{\kappa }}$ such that ${\partial }_{t}{{{{{{{{\mathcal{N}}}}}}}}}_{p}=0$. Similar calculations can be performed for effective theories away from the band top (bottom), $i\hslash {\partial }_{t}\psi =\left[E({K}_{0})+i\sqrt{\kappa }\hslash {v}_{F}y\left({\partial }_{y}-1/(2y)\right)\right]\psi$. Straightforward calculations show that ${\partial }_{t}{{{{{{{{\mathcal{N}}}}}}}}}_{p}=-\sqrt{\kappa }{v}_{F}{{{{{{{\mathcal{N}}}}}}}}$, which explains the imaginary part of the eigenenergy, ${{{{{{{\rm{Im}}}}}}}}(E)=-\sqrt{\kappa }\hslash {v}_{F}/2$.

**Fig. 2:**
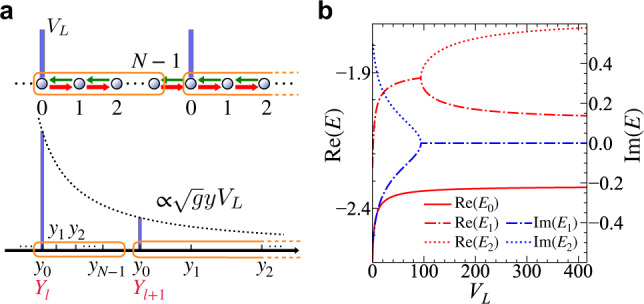
Changing boundary conditions. **a** A constant potential, *V*_*L*_, is added to the first site in each unit cell of the superlattice. The corresponding potential in the curved space depends on the position. **b** Eigenenergies for the ground state ${E}_{0}\in {\mathbb{R}}$ and the first two excited states ${E}_{1,2}\in {\mathbb{C}}$ as functions of *V*_*L*_. $\sqrt{{t}_{R}/{t}_{L}}=1.5$ and *N* = 12 are used.

Despite that *y*_0_ ≠ *y*_*N*−1_, these two edges of a hyperbolic surface can be identified in mathematics, since the solutions under PBC exist, as we previously discussed. In physical systems, such PBC can also be realized. In fact, the boundary condition can be continuously tuned. An onsite energy offset, *V*_*L* _≥ 0, in one of the lattice sites of the HN model continuously changes PBC to OBC once *V*_*L*_ increase from 0 to *∞*. We consider a superlattice of a lattice spacing of *Nd*, whose unit cell is a HN chain, as shown in Fig. 2a. Figure 2b shows eigenenergies as functions of *V*_*L*_. Similarly, an external potential can be added to the Poincaré half-plane,

11 $${V}_{\delta }=d{V}_{L}\sqrt{\kappa }y\mathop{\sum}\limits_{l}\delta (y-{Y}_{l}),$$

where ${Y}_{l}={y}_{0}{e}^{Nl\sqrt{\kappa }d}$ is the lattice site of the superlattice. The *y*-dependent amplitude of the delta-functions guarantees the scale invariance and the equivalence between each section between *Y*_*l*_ and *Y*_*l*+1_. With *V*_*L*_ increasing from zero to infinity, eigenstates evolve from those under PBC to the ones under OBC. $\int\nolimits_{{Y}_{l}^{-}}^{{Y}_{l}^{+}}\sqrt{g}{{{{{{{\rm{d}}}}}}}}y{V}_{\delta } \sim 1/\sqrt{\kappa }$ sets the energy scale of the potential, such that the larger the non-Hermiticity is, the more sensitive of the system is to the boundary condition.

### Generalizations to long-range and non-uniform tunnelings

Whereas the HN model provides an illuminating example of the duality, applications of our approach to generic non-Hermitian models are straightforward. We consider

12 $$-\mathop{\sum }\limits_{m=1}^{M}{t}_{Rm}{\psi }_{n-m}-\mathop{\sum }\limits_{m=1}^{M}{t}_{Lm}{\psi }_{n+m}=E{\psi }_{n},$$

where *t*_*Rm*_ and *t*_*Lm*_ are tunneling amplitudes from the (*n* ∓ *m*)th to *n*th sites. An eigenstate under OBC in the bulk is written as *e*^*iknd*+*qnd*^, where *kd* ∈ [0, 2*π*] and *q* is real. Unlike the HN model, where $q=\ln ({t}_{R}/{t}_{L})/(2d)$ is a constant, once beyond the nearest neighbor tunnelings exist, *q* becomes a function of *k* and defines the so-called generalized Brillouin zone (BZ) in the complex plane^10,15,27,45,46^. Near any point in the generalized BZ specified by *K*_0_*d* ∈ [0, 2*π*], we define $\psi (s)={e}^{i{K}_{0}s}{e}^{q({K}_{0})s}\phi (s)$, where *ϕ*(*s*) changes slowly as a function of *s*, corresponding to small deviations of the momentum in the continuum limit. Similar to discussions about the HN model, the effective theory can be formulated straightforwardly using $\phi ({s}_{n\pm 1})=\phi ({s}_{n})\pm d{\partial }_{s}\phi \,+\frac{1}{2}{d}^{2}{\partial }_{s}^{2}\phi$. The Schrödinger equation satisfied by *ψ*(*s*) is written as

13 $$-{{{{{{{\mathcal{B}}}}}}}}({K}_{0})[{\partial }_{s}^{2}-2{{{{{{{\mathcal{A}}}}}}}}({K}_{0}){\partial }_{s}+{{{{{{{\mathcal{C}}}}}}}}({K}_{0})]\psi (s)=E\psi (s),$$

where ${{{{{{{\mathcal{A}}}}}}}}({K}_{0})$, ${{{{{{{\mathcal{B}}}}}}}}({K}_{0})$ and ${{{{{{{\mathcal{C}}}}}}}}({K}_{0})$ depend on *K*_0_, as well as *t*_*Rm*_ and *t*_*Lm*_. When only the nearest neighbor tunnelings exist, the above equation recovers Eq. (5) at *K*_0_ = 0 and ${{{{{{{\mathcal{A}}}}}}}}({K}_{0})$ becomes real and reduces to a constant imaginary vector potential $\sim \ln ({t}_{R}/{t}_{L})/(2d)$ that we have discussed in the HN model. In the most generic case, ${{{{{{{\mathcal{A}}}}}}}}({K}_{0})$ provides a complex vector potential, whose real part curves the space. Using a coordinate transformation $y={y}_{0}{e}^{2{{{{{{{{\mathcal{A}}}}}}}}}_{R}({K}_{0})s}$, where ${{{{{{{{\mathcal{A}}}}}}}}}_{R}({K}_{0})$ is the real part of ${{{{{{{\mathcal{A}}}}}}}}({K}_{0})$, a hyperbolic surface is thus obtained in the same manner as the HN model. The only difference is that *κ* now is written as $\kappa =4{{{{{{{{\mathcal{A}}}}}}}}}_{R}^{2}$ and depends on *K*_0_. Such *K*_0_-dependent curvature provides a geometric interpretation for the generalized BZ. Explicit calculations for a model including the next-nearest-neighbor interaction are given in Supplementary Materials (Fig. S1).

Whereas uniform chiral tunnelings lead to a hyperbolic surface with a constant curvature, we could also consider non-Hermitian models with non-uniform tunnelings,

14 $$-{t}_{R,n-1}{\psi }_{n-1}-{t}_{L,n}{\psi }_{n+1}=E{\psi }_{n},$$

which gives rise to inhomogeneous local curvatures. For slowly varying *t*_*R*,*n*_ and *t*_*L*,*n*_, we define $\bar{t}(s)$ and $\bar{\gamma }(s)$ such that $\bar{t}(nd)=\frac{2M{d}^{2}}{{\hslash }^{2}}\sqrt{{t}_{R,n}{t}_{L,n}}$ and $\bar{\gamma }(nd)=\sqrt{{t}_{R,n}/{t}_{L,n}}$. We introduce a slowly changing function *ϕ*(*s*) = *e*^*ν*(*s*)/2^*ψ*(*s*) with $\nu (s)=\frac{2}{d}\int\nolimits_{0}^{s}\ln (\bar{\gamma }(s^{\prime} )){{{{{{{\rm{d}}}}}}}}s^{\prime}$. This is a generalization of the uniform case, where *ν*(*nd*) reduces to a linear function of *n*, i.e., the previously discussed $n\ln (\gamma )$ in the HN model. Using the same procedure, we obtain the effective theory of Eq. (14). For instance, the non-relativistic theory is written as

15 $$\frac{{\hslash }^{2}}{2M}\left(-\frac{1}{\sqrt{g}}{\partial }_{i}{g}^{ij}\sqrt{g}{\partial }_{j}-\frac{\kappa }{4}+{V}_{c}\right){{\Psi }}(x,y)=E{{\Psi }}(x,y),$$

where ${g}_{xx}={g}_{yy}=\sqrt{g}=\bar{t}({s}_{y}){e}^{-\frac{4}{d}\int\nolimits_{0}^{{s}_{y}}\ln (\bar{\gamma }(s^{\prime} )){{{{{{{\rm{d}}}}}}}}s^{\prime} }$, *g*_*xy*_ = *g*_*yx*_ = 0, ${V}_{c}=\frac{{\hslash }^{2}}{2M{d}^{2}}\left(\frac{d}{2}{\partial }_{s}\ln \bar{\gamma }{| }_{{s}_{y}}-2\right)\bar{t}({s}_{y})$, and the position-dependent curvature is written as $\kappa (y)=\bar{t}({s}_{y})\left(4\ln {\bar{\gamma }}^{2}({s}_{y})-2d{\partial }_{s}\ln \bar{\gamma }{| }_{{s}_{y}}\right)/{d}^{2}$. In these expressions, *s*_*y*_ is obtained from $y-{y}_{0}=\int\nolimits_{0}^{{s}_{y}}{{{{{{{\rm{d}}}}}}}}s^{\prime} {e}^{\frac{2}{d}\int\nolimits_{0}^{s^{\prime} }\ln (\bar{\gamma }(s^{\prime\prime} )){{{{{{{\rm{d}}}}}}}}s^{\prime\prime} }/\bar{t}(s^{\prime} )$. The constant *κ* of a hyperbolic surface is recovered when *t*_*R*,*n*_ and *t*_*L*,*n*_ are constants. Changing *t*_*R*,*n*_ and *t*_*L*,*n*_ then tunes local curvatures. For instance, when ${t}_{R,n}=\frac{{\hslash }^{2}}{2M{d}^{2}}{e}^{-{{\Theta }}(n-{n}^{* })/(2n)}$, ${t}_{L,n}=\frac{{\hslash }^{2}}{2M{d}^{2}}{e}^{{{\Theta }}(n-{n}^{* })/(2n)}$, where Θ(*x*) is the Heaviside step function, the curvature vanishes everywhere except at a particular location, i.e., *κ* ~ *δ*(*y* − y^*^), where y^*^ = *y*_0_ + n^*^d.

In addition to one dimension, many non-Hermitian models in higher dimensions can be constructed based on the HN model. For instance, 1D HN chains can be assembled to access higher dimensional curved spaces. Whereas curved spaces in higher dimensions are, in general, more complex than those in two dimensions, inter-chain couplings can be engineered to access different higher dimensional curved spaces (see Fig. S2 of Supplementary Materials).

### Non-Hermitian realization of QHS in curved spaces

**Fig. 3:**
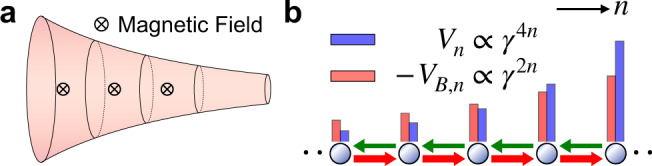
Non-Hermitian realization of QHS on hyperbolic surface. **a** A hyperbolic surface threaded by uniform magnetic fluxes. **b** An extra onsite energy in HN chain, *V*_*B*,*n*_, encapsulates the magnetic field.

The duality we established has a wide range of profound applications. For instance, Fig. 3a shows a non-Hermitian realization of QHS in curved spaces. When a particle with a charge − *e* is subjected to a uniform magnetic field, *y*^2^∇^2^ in Eq. (3) is replaced by ${y}^{2}[{({\partial }_{x}-i\frac{eB}{\hslash \kappa }\frac{1}{y})}^{2}+{\partial }_{y}^{2}]$, where we have chosen the gauge with the vector potential **A** = (−*B*/(*κy*), 0)^47^ such that *k*_*x*_ is still a good quantum number. Wavefunctions of the lowest Landau level (LLL) are written as,

16 $${\psi }_{{{{{{{{\rm{LLL}}}}}}}}}={(2{k}_{x})}^{\frac{eB}{\hslash \kappa }-\frac{1}{2}}\sqrt{\frac{{{{{{{{\mathcal{N}}}}}}}}\kappa }{{{\Gamma }}(2\frac{eB}{\hslash \kappa }-1)L}}{e}^{-{k}_{x}y+i{k}_{x}x}{y}^{\frac{eB}{\hslash \kappa }},$$

whose eigenenergies, ${E}_{LLL}=-\frac{{\hslash }^{2}\kappa }{8M}+\frac{\hslash eB}{2M}$, are independent of *k*_*x*_, manifesting the degeneracy of the Landau levels. In the dual non-Hermitian systems, a finite magnetic field corresponds to an extra onsite potential in Eq. (4), *V*_*n*_ → *V*_*n*_ + *V*_*B*,*n*_, where ${V}_{B,n}=-ba{\gamma }^{2n}\sqrt{{t}_{L}{t}_{R}}$, as shown in Fig. 3b. The dimensionless *b* characterizing the strength of *V*_*B*,*n*_ relative to *V*_*n*_ is written as

17 $$b=eB{d}^{2}/(\hslash \ln | \gamma | ),$$

where ${y}_{0}{k}_{x}=a{(2\ln | \gamma | )}^{-1}$ has been used. The magnetic flux density, ${\rho }_{\phi }=eB/(2\pi \hslash )=\frac{\ln | \gamma | }{2\pi {d}^{2}}b$, is thus determined by the ratio of *V*_*B*,*n*_ to *V*_*n*_. For a given *B*, a finite *k*_*x*_ shifts the position of the minimum of the total onsite potential *V*_*n*_ + *V*_*B*,*n*_, similar to the well-known result of flat spaces where *k*_*x*_ determines the location of the minimum of the potential in the Landau gauge.

A complete description of QHS requires its gravitational responses in curved spaces. For instance, the particle density, *ρ*, depends on the local curvature^36^,

18 $$\rho =\nu {\rho }_{\phi }-\kappa /(4\pi ),$$

where *ν* is the filling factor. For integer QHS, *ν* = 1 and Eq. (18) for a hyperbolic surface can be straightforwardly proved using Eq. (16) (Supplementary Materials). The counterpart of Eq. (18) in the non-Hermitian lattice is

19 $$| \gamma {| }^{2n}\int {{{{{{{\rm{d}}}}}}}}a{{{{{{{{\mathcal{N}}}}}}}}}_{n}(a,b)=b\ln (| \gamma | )-2{\ln }^{2}(| \gamma | ),$$

where ${{{{{{{{\mathcal{N}}}}}}}}}_{n}(a,b)=| \gamma {| }^{-2n}{\psi }_{n}^{* }{\psi }_{n}$ is the particle number at lattice site *n* in the non-Hermitian system. Mapping the magnetic flux, *eB*/(2*πℏ*), to the ratio between onsite potentials, $b\ln (| \gamma | )/(2\pi {d}^{2})$, Eq. (18) and Eq. (19) are equivalent. The dependence of densities of QHS on curvatures is thus readily detectable using this non-Hermitian realization. An alternative scheme is to implement a 2D non-Hermitian lattice model as illustrated in Fig. S3 of Supplementary Materials, which serves as a non-Hermitian generalization of the Harper-Hofstadter Hamiltonian^48^.

## Discussion

In parallel to accessing curved spaces using non-Hermitian systems, experimentalists could also use curved spaces to study non-Hermitian physics^32,33,35^. In conventional understandings, non-Hermiticity arises when dissipations exist. While dissipations have been engineered in certain apparatuses to deliver desired non-Hermitian Hamiltonians^1,6,7^, in other platforms, such engineering might be more difficult and sometimes experimentalists may have to use indirect means such as simulating non-Hermitian quantum walks^2,8,27^. Our results show that many non-Hermitian Hamiltonians are readily accessible using existing curved spaces. For instance, hyperbolic surfaces that have been created in laboratories could be used to realize the HN model directly. In particular, in contrast to current schemes used in the study of non-Hermitian physics, this method does not require engineering losses or gains. It thus provides a conceptually different protocol to access non-Hermiticity without dissipations.

The duality we have found provides insightful perspectives for both the studies of non-Hermitian physics and curved spaces. As the curvature depends on the non-Hermiticity even when the separation between any two points in the system is not physically distorted, our conventional understandings of distance may need to be reformed. We hope that our work will stimulate more interest in studying deep connections between non-Hermitian physics and curved spaces.

## Supplementary information

Supplementary Information

## Data Availability

Numerical data for the presented plots are available from the authors upon request.

## Code availability

Computer codes for generating the figures presented are available from the authors upon request.
