# Supplementary material for: Curving the space by non-Hermiticity
Source: Nat Commun. 2022 Apr 21;13:2184. doi: 10.1038/s41467-022-29774-8 (PMC9023518; doi:10.1038/s41467-022-29774-8)
Supplement: Supplementary file 1 — Supplementary Information [file 41467_2022_29774_MOESM1_ESM.pdf]

Supplementary Information to “Curving the space by non-Hermiticity”

Chenwei Lv,[1] Ren Zhang,[2,1] Zhengzheng Zhai,[1] and Qi Zhou[1,3]

[1] *Department of Physics and Astronomy, Purdue University, West Lafayette, IN, 47907, USA*
[2] *School of Science, Xi'an Jiaotong University, Xi'an, Shaanxi 710049*
[3] *Purdue Quantum Science and Engineering Institute, Purdue University, West Lafayette, IN 47907, USA*

(Dated: March 8, 2022)

Ricci scalar curvature and mean curvature

In the main text, we considered an intrinsic curved space where the quantum particle couples to the Ricci scalar curvature $R_{\rm Ric}$. Whereas the choice of the coupling constant is not unique [1], we have adopted the one used in Ref. [2]. In 2D, the Ricci scalar curvature relates to the Gaussian curvature $-\kappa$ through $-\kappa = R_{\rm Ric}/2$. We therefore obtain a potential term proportional to the Gaussian curvature $-\kappa$.

In addition to intrinsic curved spaces, an alternative approach is to consider curved spaces embedded in a higher dimensional flat space. This requires applying physical constraints in the higher dimensional space such that particles can only move in a certain subspace that is curved. The physical constraints thus induce an extra potential that also depends on the mean curvature [3]. Considering the pseudosphere, which is an embedded hyperbolic surface in 3D as shown in Eq. (9) of the main text, its first and second fundamental forms are written as

$$\mathbf{g} = \frac{1}{\kappa}\begin{pmatrix} \tanh^2(\eta) & 0 \\ 0 & \operatorname{sech}^2(\eta)\end{pmatrix}, \quad \mathbf{h} = \frac{1}{\sqrt{\kappa}}\begin{pmatrix} -\operatorname{sech}(\eta)\tanh(\eta) & 0 \\ 0 & \operatorname{sech}(\eta)\tanh(\eta)\end{pmatrix}, \tag{S1}$$

respectively. The mean curvature and Gaussian curvature become

$$\begin{aligned} K_{\rm mean} &= \frac{1}{2g}(g_{11}h_{22} + g_{22}h_{11} - 2g_{12}h_{12}) = \frac{1}{4}\sqrt{\kappa}(-3+\cosh(2\eta))\operatorname{csch}(\eta), \\ K_{\rm Gaussian} &= \frac{1}{g}\det(\mathbf{h}) = -\kappa, \end{aligned} \tag{S2}$$

respectively. The Schrödinger equation that describes quantum particles confined to this hyperbolic surface through an external potential along the normal direction contains a surface potential [3]

$$V_S(\eta) = -\frac{\hbar^2}{2M}\left(K_{\rm mean}^2 - K_{\rm Gaussian}\right) = -\frac{\hbar^2\kappa}{2M}\left(\frac{1}{16}(-3+\cosh(2\eta))^2\operatorname{csch}^2(\eta) + 1\right). \tag{S3}$$

Transforming to the s-coordinate, it amounts to

$$V_S(s) = -\frac{\hbar^2\kappa}{8M}\frac{e^{4\sqrt{\kappa}s}}{e^{2\sqrt{\kappa}s}-1}. \tag{S4}$$

We have chosen the constant $r_0 = y_0$ such that $\eta(s=0) = 0$. V_S only contributes to an extra potential in the curved space once applying our duality. Correspondingly, an on-site potential $V_{S,n} = V_S(nd)$ introduced in the non-Hermitian model allows us to simulate embedded curved spaces.

The HN model with the next-nearest-neighbor hopping

In the presence of next-nearest-neighbor hoppings, the non-Hermitian model is written as

$$-t_{R2}\psi_{n-2} - t_{L2}\psi_{n+2} - t_{R1}\psi_{n-1} - t_{L1}\psi_{n+1} = E\psi_n. \tag{S5}$$

A generic solution to this Schrödinger equation is written as β^n, with eigenenergy $E(\beta) = -(t_{R2}\beta^{-2} + t_{L2}\beta^2 + t_{R1}\beta^{-1} + t_{L1}\beta)$. For the open boundar y condition, $\psi_{n<0} = \psi_{n>N-1} = 0$, the Schrödinger equation formally becomes different near the edges,

$$\begin{aligned} -t_{L2}\psi_2 - t_{L1}\psi_1 = E\psi_0, &\quad -t_{L2}\psi_3 - t_{R1}\psi_0 - t_{L1}\psi_2 = E\psi_1, \\ -t_{R2}\psi_{N-3} - t_{R1}\psi_{N-2} = E\psi_{N-1}, &\quad -t_{R2}\psi_{N-4} - t_{R1}\psi_{N-3} - t_{L1}\psi_{N-1} = E\psi_{N-2}. \end{aligned} \tag{S6}$$

Alternatively, the boundary condition can be written as $\psi_{-1} = \psi_{-2} = \psi_N = \psi_{N+1} = 0$.

For a given complex energy E, $t_{L2}\beta^4 + t_{L1}\beta^3 + E\beta^2 + t_{R1}\beta + t_{R2} = 0$ has four solutions, denoted by $|\beta_1| \leq |\beta_2| \leq |\beta_3| \leq |\beta_4|$. A specific solution to boundary conditions Eq. (S6) is written as

$$\psi_n = c_1\beta_1^n + c_2\beta_2^n + c_3\beta_3^n + c_4\beta_4^n, \tag{S7}$$

It is known that $|\beta_1| \leq |\beta_2| = |\beta_3| \leq |\beta_4|$ is required to satisfy the boundary condition when $N \to \infty$. This leads to the definition of the Generalized Brillouin Zone (GBZ) [4–6]. As such, β_2^n and β_3^n are dominant in the bulk. An eigenstate is depicted in Fig. S1.

Using β_2 and β_3, we could formulate the effective theory in the bulk. We denote β_2 by $e^{(q+iK_0)d}$ and β_3 by $e^{(q+iK_1)d}$ where q, K_0 and K_1 are real. Using the same method discussed in the main text, we define $\sqrt{d}\psi(s_n) \equiv \psi_n$, $\psi_0(s) = \phi_0(s)e^{iK_0 s}e^{qs}$ such that $\phi_0(s)$ is slowly varying with changing s, the Schrödinger equation for ψ_0 in the continuum limit is written as

$$-\mathcal{B}(K_0)\left[\partial_s^2 - 2\mathcal{A}(K_0)\partial_s + \mathcal{C}(K_0)\right]\psi_0(s) = E\psi_0(s), \tag{S8}$$

where

$$\begin{aligned}
\mathcal{B}(K_0) &= \sum_{n=1,2}\left(t_{Rn}\beta_2^{-n} + t_{Ln}\beta_2^n\right)\frac{n^2d^2}{2},\\
\mathcal{A}(K_0) &= (iK_0+q) - \frac{\sum_{n=1,2}\left(-t_{Rn}\beta_2^{-n} + t_{Ln}\beta_2^n\right)n}{d\sum_{n=1,2}\left(t_{Rn}\beta_2^{-n} + t_{Ln}\beta_2^n\right)n^2},\\
\mathcal{C}(K_0) &= (iK_0+q)^2 - (iK_0+q)\frac{2\sum_{n=1,2}\left(-t_{Rn}\beta_2^{-n} + t_{Ln}\beta_2^n\right)n}{d\sum_{n=1,2}\left(t_{Rn}\beta_2^{-n} + t_{Ln}\beta_2^n\right)n^2} + \frac{2\sum_{n=1,2}\left(t_{Rn}\beta_2^{-n} + t_{Ln}\beta_2^n\right)}{d^2\sum_{n=1,2}\left(t_{Rn}\beta_2^{-n} + t_{Ln}\beta_2^n\right)n^2}.
\end{aligned} \tag{S9}$$

Since q has been determined by K_0 in GBZ, $\mathcal{A}(K_0)$, $\mathcal{B}(K_0)$, $\mathcal{C}(K_0)$ are known. A coordinate transformation $y/y_0 = e^{2s\mathcal{A}_R}$ leads to

$$-\mathcal{B}(K_0)\left[4\mathcal{A}_R(K_0)^2y^2\partial_y^2 - 4i\mathcal{A}_I(K_0)\mathcal{A}_R(K_0)y\partial_y + \mathcal{C}(K_0)\right]\psi_0(y) = E\psi_0(y), \tag{S10}$$

where $\mathcal{A}_R$ and $\mathcal{A}_I$ denotes the real and imaginary part of $\mathcal{A}$, respectively. Similarly, using β_3, we can define $\psi_1(s) = \phi_1(s)e^{iK_1 s}e^{qs}$ and obtain,

$$-\mathcal{B}(K_1)\left[4\mathcal{A}_R(K_1)^2y^2\partial_y^2 - 4i\mathcal{A}_I(K_1)\mathcal{A}_R(K_1)y\partial_y + \mathcal{C}(K_1)\right]\psi_1(y) = E\psi_1(y). \tag{S11}$$

Both equations describe a Poincaré half-plane, which is subject to a real constant vector potential, $\vec{A}_{j=0,1} \sim \mathcal{A}_I(K_j)$, if $\mathcal{A}_I(K_j) \neq 0$. $\mathcal{A}_R(K_j)$ determines the curvature, $-\kappa_j = -4\mathcal{A}_R^2(K_j)$, and the mass is given by $\frac{\hbar^2}{2M} = \mathcal{B}(K_j)$.

When t_{L2} and t_{R2} vanish, we obtain $qd = \ln\left(\sqrt{t_{R1}/t_{L1}}\right)$, and Eq.(S10) reduces to,

$$-\sqrt{t_{L1}t_{R1}}\cos(K_0d)\left[\left(2qdy\left(\partial_y - \frac{1}{2y}\right) - iK_0d\right)^2 + 4qd\tan(K_0d)iy\left(\partial_y - \frac{1}{2y}\right) + 2K_0d\tan(K_0d) + 2\right]\psi_0(y) = E\psi_0(y). \tag{S12}$$

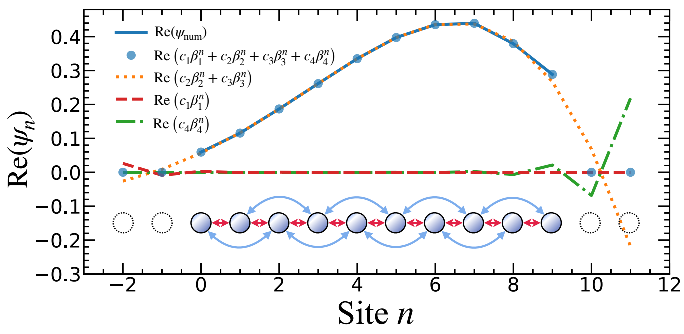

FIG. S1. An eigenstate of the HN model with the next nearest neighbor tunnelings. We use $N = 10$, $t_{L1} = 0.5t_{R1}$, $t_{R2} = 0.6t_{R1}$, and $t_{L2} = 0.4t_{R1}$. The real parts of wavefunction are shown and the imaginary parts vanish. The numerical solution is extrapolated to the lattice sites −1, −2, 10 and 11 by $\psi_{\text{num}} = c_1\beta_1^n + c_2\beta_2^n + c_3\beta_3^n + c_4\beta_4^n$. Here, $c_2\beta_2^n + c_3\beta_3^n$ forms a standing wave, $c_1\beta_1^n$ and $c_4\beta_4^n$ are localized at the boundary to fulfill the boundary condition.

For $K_0 d = 0, \pi$, we recover the non-relativistic theory in the main text. $\mathcal{A}_R^2(K_0)$ also reduces to q^2 such that $-\kappa = -4q^2$, as expected.

For $K_0 d \neq 0, \pi$, the first term in the square brackets of the above equation corresponds to high order corrections to the energy and the effective theory becomes

$$-\sqrt{t_{L1}t_{R1}}\cos(K_0 d)\left[4qd\tan(K_0 d) iy\left(\partial_y - \frac{1}{2y}\right) + 2K_0 d\tan(K_0 d) + 2\right]\psi_0(y) = E\psi_0(y). \tag{S13}$$

Similarly, we obtain

$$-\sqrt{t_{L1}t_{R1}}\cos(K_1 d)\left[4qd\tan(K_1 d) iy\left(\partial_y - \frac{1}{2y}\right) + 2K_1 d\tan(K_1 d) + 2\right]\psi_1(y) = E\psi_1(y). \tag{S14}$$

Both equations describe effective theories for particles with linear dispersions on a Poincaré half-plane, $\hat{H} = -v_f\sqrt{\kappa}\frac{1}{2}(y\hat{p}_y + \hat{p}_y y) = i\hbar v_f\sqrt{\kappa}y\left(\partial_y - \frac{1}{2y}\right)$, where v_f is the Fermi velocity and $\hat{p}_y = -i\hbar\left(\partial_y - \frac{1}{y}\right)$, $\hat{p}_x = -i\hbar\partial_x$. Comparing this Hamiltonian with that in Eq. (S13,S14), we obtain

$$-\kappa = -4q^2, \quad v_{f,j} = -2\sqrt{t_{L1}t_{R1}}d\sin(K_j d)/\hbar, \quad j = 0, 1, \tag{S15}$$

which recover the results in the main text, as expected.

Coupled HN chains

The lattice model with HN chains coupled vertically is illustrated in Fig. S2a, which reads

$$-t\Psi_{n,m+1} - t\Psi_{n,m-1} - t_R\Psi_{n-1,m} - t_L\Psi_{n+1,m} = E\Psi_{n,m}. \tag{S16}$$

We define $\Psi(s,z)$ such that $\Psi(s_n, z_m) \equiv \Psi_{n,m}/d$, and $\Psi(s,z) = \Phi(s,z)e^{qs}e^{iK_0 s}e^{ik_{z0}z}$, where Φ is slowly varying. We have applied periodical boundary condition along the z-direction. Near the band bottom ($K_0 = 0, k_{z0} = 0$), substituting Ψ to Eq. (S16) and using the Taylor expansion of $\Phi(s,z)$, we obtain

$$-\left[\left(2t + t_R e^{-qd} + t_L e^{qd}\right) + \left(-t_R e^{-qd} + t_L e^{qd}\right)d\partial_s + \frac{d^2}{2}\left(t_R e^{-qd} + t_L e^{qd}\right)\partial_s^2 + td^2\partial_z\right]\Phi(s,z) = E\Phi(s,z) \tag{S17}$$

A generic solution is written as $\Phi = e^{ik_s s}e^{ik_z z}$. To ensure the open boundary condition in s-direction and obtain an effective theory near ($K_0 = 0, k_{z0} = 0$), we require $E(k_s, 0) = E(-k_s, 0)$, which leads to $-t_R e^{-qd} + t_L e^{qd} = 0$. The solution gives $q = \ln\left(\sqrt{t_R/t_L}\right)/d$. For a finite k_z, the solution to Eq.(S17) that satisfies the specified boundary conditions is written as $e^{ik_z z}\sin(k_s s)$. In other words, q and the resultant curvature, is independent of k_z. After a coordinate transformation $\frac{y}{y_0} = e^{2qs}$, Eq. (S17) is written as

$$-\left[2\left(t + \sqrt{t_L t_R}\right) + \sqrt{t_L t_R}d^2\kappa\left(y^2\partial_y^2 + \frac{1}{4}\right) + td^2\partial_z^2\right]\Psi(y,z) = E\Psi(y,z), \tag{S18}$$

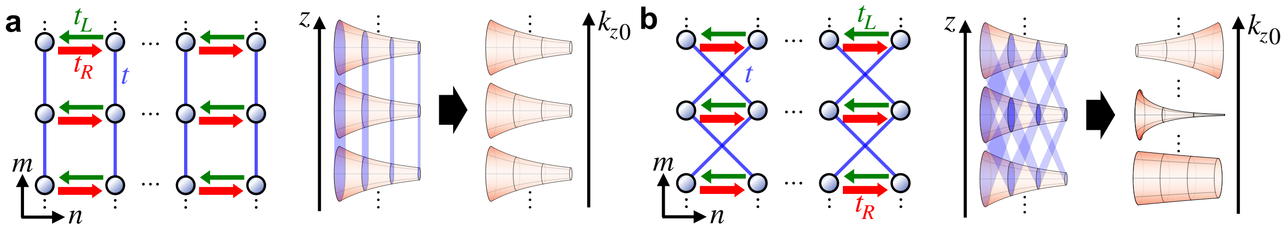

FIG. S2. A set of coupled HN chains is dual to a 3D curved space. (a) The curvature of each decoupled surface remains unchanged by the vertical inter-chain couplings. (b) Inter-chain couplings fundamentally influence each curved surface, and the curvature becomes energy-dependent.

where $\kappa = 4\ln^2\left(\sqrt{\frac{t_R}{t_L}}\right)/d^2$.

In contrast, the model in Fig. S2b reads

$$-t\Psi_{n-1,m-1} - t\Psi_{n+1,m-1} - t\Psi_{n-1,m+1} - t\Psi_{n+1,m+1} - t_R\Psi_{n-1,m} - t_L\Psi_{n+1,m} = E\Psi_{n,m}. \tag{S19}$$

The slowly varying $\Phi(s,z)$ satisfies

$$\begin{aligned}&-\Big[\left(2te^{-qd} + 2te^{qd} + t_R e^{-qd} + t_L e^{qd}\right) + \left(-2te^{-qd} + 2te^{qd} - t_R e^{-qd} + t_L e^{qd}\right) d\partial_s \\ &\quad + \left(2te^{-qd} + 2te^{qd} + t_R e^{-qd} + t_L e^{qd}\right)\frac{1}{2}d^2\partial_s^2 + 2t\left(e^{qd} + e^{-qd}\right)\frac{1}{2}d^2\partial_z^2 + t\left(e^{qd} - e^{-qd}\right)d^3\partial_z^2\partial_s\Big]\Phi(s,z) = E\Phi(s,z).\end{aligned} \tag{S20}$$

For the effective theory near $(K_0 = 0, k_{z0} = 0)$, the open boundary condition in s-direction requires $E(k_s, 0) = E(-k_s, 0)$ and $-2te^{-qd} + 2te^{qd} - t_R e^{-qd} + t_L e^{qd} = 0$. This provides us with $q = \frac{1}{d}\ln\left(\sqrt{\frac{t_R+2t}{t_L+2t}}\right)$, and

$$-\left[2\tilde{t} + \tilde{t}d^2\partial_s^2 + \frac{t}{\tilde{t}}(4t + t_L + t_R)d^2\partial_z^2 + \frac{t}{\tilde{t}}(t_R - t_L)d^3\partial_z^2\partial_s\right]\Phi(s,z) = E\Phi(s,z), \tag{S21}$$

where $\tilde{t} = \sqrt{(t_R + 2t)(t_L + 2t)}$. When k_z is finite, the solution to Eq.(S21) reads $\Phi = e^{ik_z z}\sin(k_s s)e^{\left(\frac{t(t_R - t_L)}{2\tilde{t}^2}k_z d\right)k_z s}$. The extra exponential function gives rise to a k_z-dependent $q(k_z) = \frac{1}{d}\ln\left(\sqrt{\frac{t_R+2t}{t_L+2t}}\right) + \frac{td(t_R - t_L)}{2\tilde{t}^2}k_z^2$. After a coordinate transformation $\frac{y}{y_0} = e^{2qs}$ and $\Phi(s,z) = \Psi(s,z)e^{-qs}$, Eq. (S21) is written as

$$-\tilde{t}d^2\left[\frac{2}{d^2} + \kappa_c\left(y^2\partial_y^2 + \frac{1}{4}\right) + \frac{t(t_R - t_L)}{\tilde{t}^2}\partial_z^2\left(\frac{4t + t_R + t_L}{t_R - t_L} + d\sqrt{\kappa_c}\left(y\partial_y - \frac{1}{2}\right)\right)\right]\Psi(y,z) = E\Psi(y,z), \tag{S22}$$

where $\kappa_c = \ln^2\left(\frac{t_R+2t}{t_L+2t}\right)/d^2$. A finite k_z modifies the curvature, $\kappa_c(k_z) = \frac{1}{d^2}\ln^2\left(\frac{t_R+2t}{t_L+2t}\right) + \frac{2t(t_R - t_L)}{\tilde{t}^2}\log\left(\frac{t_R+2t}{t_L+2t}\right)k_z^2 + \mathcal{O}((k_z d)^4)$.

Whereas the above discussions apply to the band bottom, the effective theory can be formulated at any energies. In the lattice models, we write $\Psi_{n,m} = \psi_n e^{ik_{z0}md}$, and obtain

$$-2t\cos(k_{z0}d)\psi_n - t_R\psi_{n-1} - t_L\psi_{n+1} = E\psi_n, \tag{S23}$$

and

$$-(t_R + 2t\cos(k_{z0}d))\psi_{n-1} - (t_L + 2t\cos(k_{z0}d))\psi_{n+1} = E\psi_n, \tag{S24}$$

for Eq. (S16) and (S19), respectively. Therefore, for each k_{z0}, we have a dual model in the curved space. Especially, for Eq. (S19), we obtain a k_{z0}-dependent HN model and the corresponding curved space can be derived in the same manner as in the main text, where $\kappa = \ln^2\left(\left|\frac{t_R+2t\cos(k_{z0}d)}{t_L+2t\cos(k_{z0}d)}\right|\right)/d^2$, which is k_{z0} dependent. It reduces to κ_c if we take the limit of $k_{z0}d \to 0$.

Gravitational responses of quantum Hall states in hyperbolic spaces

The normalized wavefunction at the lowest Landau level on a Poincaré half-plane is written as

$$\Psi_{\rm LLL}(x,y) = 2^{\alpha-1/2}k_x^{\alpha-1/2}\sqrt{\frac{\kappa\mathcal{N}}{\Gamma(2\alpha-1)L}}e^{ik_x x}e^{-k_x y}y^\alpha, \tag{S25}$$

where $\Gamma(x)$ is the Gamma function $\alpha = \frac{eB}{\hbar\kappa} > 0$, and $k_x = 2\pi n/L$. The particle density at unit filling becomes

$$\rho(x,y) = \frac{1}{\mathcal{N}}\sum_{k_x}|\Psi_{\rm LLL}|^2 = \sum_{n=1}^{\infty}\left(\frac{4\pi n}{L}\right)^{2\alpha-1}e^{-\frac{4\pi n}{L}y}\frac{\kappa y^{2\alpha}}{\Gamma(2\alpha-1)L} = \left(\frac{4\pi}{L}\right)^{2\alpha-1}\frac{\kappa y^{2\alpha}}{\Gamma(2\alpha-1)L}{\rm Li}_{1-2\alpha}(e^{-\frac{4\pi}{L}y}), \tag{S26}$$

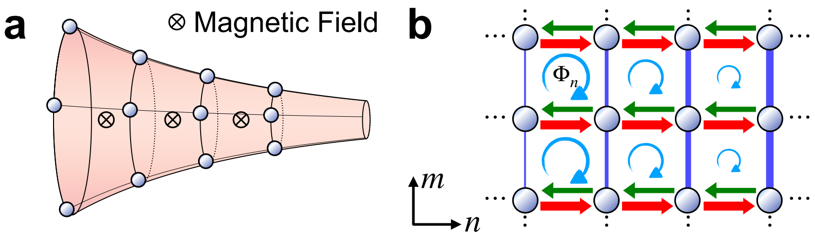

FIG. S3. A realization of hyperbolic surface threaded by uniform magnetic fluxes using 2D non-Hermitian lattices. Both vertical couplings and the magnetic flux per plaquette vary exponentially with changing n since the area of plaquettes changes exponentially on the hyperbolic surface.

where $\mathrm{Li}_k(z)=\sum_{n=1}^{\infty} z^n/n^k$ is the polylogarithm function of order k. The summation is only taken from $n=1$ to ∞ since the wavefunction is not normalizable and Landau levels do not exist for $n\le 0$. In the thermodynamic limit,

$$\lim_{L\to\infty}\rho(x,y)=(4\pi)^{-1}\frac{\Gamma(2\alpha)\kappa}{\Gamma(2\alpha-1)}=\frac{eB}{2\pi\hbar}-\frac{\kappa}{4\pi}. \tag{S27}$$

We have used $\mathrm{Li}_k(e^w)=\Gamma(1-k)(-w)^{k-1}+\sum_{j=0}^{\infty}\frac{\zeta(k-j)}{j!}w^j$, which is valid for $|w|<2\pi$ and $k\notin\mathbb{N}^+$. $\zeta(x)$ denotes the zeta function.

A non-Hermitian generalization of the Harper-Hofstadter model

We consider the following 2D non-Hermitian lattice model,

$$-t_R\Psi_{n-1,m}-t_L\Psi_{n+1,m}-\tilde{a}^2\sqrt{t_Lt_R}\gamma^{4n}\left(e^{i\theta_n}\Psi_{n,m-1}+e^{-i\theta_n}\Psi_{n,m+1}-2\Psi_{n,m}\right)=E\Psi_{n,m} \tag{S28}$$

where $\tilde{a}$ determines strengths of vertical tunnelings, $\theta_n=\tilde{b}\gamma^{-2n}/(2\tilde{a})$, and $\tilde{b}$ controls flux per plaquette. As shown in Fig. S3b, Eq.(S28), a non-Hermitian generalization of the Harper-Hofstadter Hamiltonian [7], corresponds to discretizing a hyperbolic surface in both directions. Eq.(S28) recovers the continuous model in the limit of small magnetic flux per plaquette, as we show below. We write $\Psi_{n,m}=\psi_n e^{ik_xmd}$, Eq.(S28) becomes

$$-t_R\psi_{n-1}-t_L\psi_{n+1}-2\tilde{a}^2\sqrt{t_Lt_R}\gamma^{4n}\left(-1+\cos\left(\frac{\tilde{b}}{2\tilde{a}}\gamma^{-2n}-k_xd\right)\right)\psi_n=E\psi_n. \tag{S29}$$

The magnetic flux in each plaquette is proportional to $\frac{\tilde{b}}{\tilde{a}}$. When $k_xd\ll 1$ and $\frac{\tilde{b}}{\tilde{a}}\ll 1$, we obtain

$$-t_R\psi_{n-1}-t_L\psi_{n+1}+\tilde{a}^2\sqrt{t_Lt_R}\gamma^{4n}\left(\frac{\tilde{b}}{2\tilde{a}}\gamma^{-2n}-k_xd\right)^2\psi_n=E\psi_n. \tag{S30}$$

In the low-energy limit, the continuous model is written as

$$-\sqrt{t_Rt_L}d^2\left(\partial_s-\frac{\ln(\gamma)}{d}\right)^2\psi+\sqrt{t_Lt_R}d^2\tilde{a}^2e^{4s\ln(\gamma)/d}\left(\frac{\tilde{b}}{2\tilde{a}d}e^{-2s\ln(\gamma)/d}-k_x\right)^2\psi=E\psi. \tag{S31}$$

Replacing k_x by $-i\partial_x$ and defining $\frac{y}{\tilde{a}d/(2\ln(\gamma))}=e^{2s\ln(\gamma)/d}$ yield the Schrödinger equation of a charged particle on the Poincaré half-plane,

$$-4\ln(\gamma)^2\sqrt{t_Lt_R}\left[y^2\left(\partial_y^2+\left(\partial_x-i\frac{\tilde{b}}{4\ln(\gamma)y}\right)^2\right)+\frac{1}{4}\right]\psi=E\psi. \tag{S32}$$

We can therefore identify the mass term $\frac{\hbar^2}{2M}=\sqrt{t_Lt_R}d^2$, curvature $-\kappa=-4\ln(\gamma)^2/d^2$, and the magnetic field $B=\frac{\hbar}{e}\frac{\tilde{b}\ln(\gamma)}{d^2}$. Adding interactions to this lattice model could potentially allow experimentalists to access fractional QHS with $\nu\neq 1$ or their counterparts of fractional Chern insulators in curved spaces.

[1] S. M. Carroll, Spacetime and Geometry (Cambridge University Press, 2019).
[2] M. C. Gutzwiller, Stochastic behavior in quantum scattering, Physica D: Nonlinear Phenomena **7**, 341 (1983).
[3] R. C. T. da Costa, Quantum mechanics of a constrained particle, Phys. Rev. A **23**, 1982 (1981).
[4] S. Yao and Z. Wang, Edge states and topological invariants of non-Hermitian systems, Phys. Rev. Lett. **121**, 086803 (2018).
[5] K. Yokomizo and S. Murakami, Non-Bloch band theory of non-Hermitian systems, Phys. Rev. Lett. **123**, 066404 (2019).
[6] Z. Yang, K. Zhang, C. Fang, and J. Hu, Non-Hermitian bulk-boundary correspondence and auxiliary generalized Brillouin zone theory, Phys. Rev. Lett. **125**, 226402 (2020).
[7] D. R. Hofstadter, Energy levels and wave functions of Bloch electrons in rational and irrational magnetic fields, Phys. Rev. B **14**, 2239 (1976).
